# Predictive value of the triglyceride-glucose index for adverse clinical outcomes in chronic kidney disease

**DOI:** 10.3389/fendo.2025.1656800

**Published:** 2026-01-21

**Authors:** Jeong-Yeun Lee, Su Jin Jeong, Jin Sug Kim, Kyung Hwan Jeong, Hyeon Seok Hwang

**Affiliations:** 1 Division of Nephrology, Department of Internal Medicine, College of Medicine, Kyung Hee University, Kyung Hee University Medical Center, Seoul, Republic of Korea; 2 Statistics Support Part, Medical Science Research Institute, Kyung Hee University, Seoul, Republic of Korea

**Keywords:** cardiovascular event, chronic kidney disease, kidney failure, mortality, triglyceride-glucose index

## Abstract

**Introduction:**

The triglyceride-glucose (TyG) index has been proposed as a reliable marker of insulin resistance. However, clinical significance of the TyG index in patients with chronic kidney disease (CKD) remains unclear.

**Methods:**

A total of 47,592 individuals with CKD who underwent nationwide health examinations between 2012 and 2015 were enrolled. Patients were classified into four quartiles based on their TyG index levels. The association between the TyG index and cardiovascular (CV) events, progression to end-stage kidney disease (ESKD), and all-cause mortality was evaluated.

**Results:**

A higher TyG index was associated with an increased risk of CV events. Compared with patients in the lowest TyG index quartile, hazard ratios (HRs) and 95% confidence intervals (CIs) for CV events were 1.08 (1.00–1.17), 1.13 (1.04–1.22), and 1.39 (1.28–1.51) for the second, third, and fourth quartiles, respectively. The risk of progression to ESKD also increased with higher TyG quartiles, with adjusted HRs (95% CIs) of 1.22 (1.08–1.38), 1.35 (1.18–1.54), and 1.86 (1.65–2.10) across increasing TyG index quartiles. The risk of all-cause mortality was similarly elevated in patients in the highest TyG index quartile. Furthermore, the risks of ESKD progression and all-cause mortality across different levels of kidney function was more pronounced with increasing TyG index levels (all P for interaction< 0.001).

**Conclusion:**

TyG index is a valuable predictor of CV event, progression to ESKD, and all-cause mortality in patients with CKD, and it significantly interacted with kidney function in relation to the risks of ESKD and mortality.

## 1 Introduction

Insulin resistance, characterized by decreased tissue responsiveness to insulin, leads to hyperinsulinemia and glucose intolerance. This condition is widely recognized as being associated with various pathological conditions and diseases (1, 2). Several studies have reported that insulin resistance is common among patients with chronic kidney disease (CKD) and may be present even in the early stage of CKD with preserved estimated glomerular filtration rate (eGFR) (3, 4). Approximately 50% of patients with CKD exhibit insulin resistance, and its prevalence increases as renal function declines (5). This observation has important clinical implications, as insulin resistance is strongly associated with increased cardiovascular (CV) risk in the general population, and patients with CKD exhibit an even greater CV burden (6–8).

Various methods have been developed to directly or indirectly assess insulin resistance, including the homeostatic model assessment for insulin resistance (HOMA-IR) and the hyperinsulinemic-euglycemic clamp technique. However, their clinical application is limited by the cost and accessibility of insulin testing. The triglyceride-glucose (TyG) index, derived from fasting triglyceride and glucose concentrations, has emerged as a novel marker for estimating insulin resistance (9). Previous studies have reported that a higher TyG index is associated with an increased risk of CV events and mortality in the general population (10). In addition, growing evidence supports that a higher TyG index may be related to renal dysfunction and proteinuria (11). Moreover, it has been shown that the TyG index effectively reflects insulin resistance in patients with CKD (12). However, despite the high prevalence of insulin resistance and CV risk in this population, the association between the TyG index and adverse clinical outcomes in patients with CKD remains insufficiently characterized. Additionally, the relationship between the TyG index and kidney function in predicting CV events, progression to end-stage kidney disease (ESKD), and all-cause mortality remains unclear.

Using the National Health Insurance Service database, this study evaluates the association between the TyG index and major clinical outcomes, including CV events, ESKD progression, and all-cause mortality, in patients with CKD. This investigation aims to elucidate the clinical significance of the TyG index in this population.

## 2 Materials and methods

### 2.1 Data sources

This nationwide cohort study was conducted using the National Health Information Database (NHID). The public healthcare system in South Korea operates under the National Health Insurance Service (NHIS), a universal and compulsory single-insurer system. The NHIS forms the public health database of NHID and includes a comprehensive range of information, such as demographic data, diagnoses (coded using the 10th International Classification of Diseases [ICD-10]), insured medical services, and laboratory data of the general population (13). In addition, all insured individuals are required to undergo annual or biannual National Health Screening programs. Healthcare utilization data were integrated with health screening records to constitute a study cohort. This study was approved by the Institutional Review Board (KHUH 2021-11-056), and the use of the NHIS database (NHIS-2023-1-380) was authorized.

### 2.2 Study population

**Figure 1:**
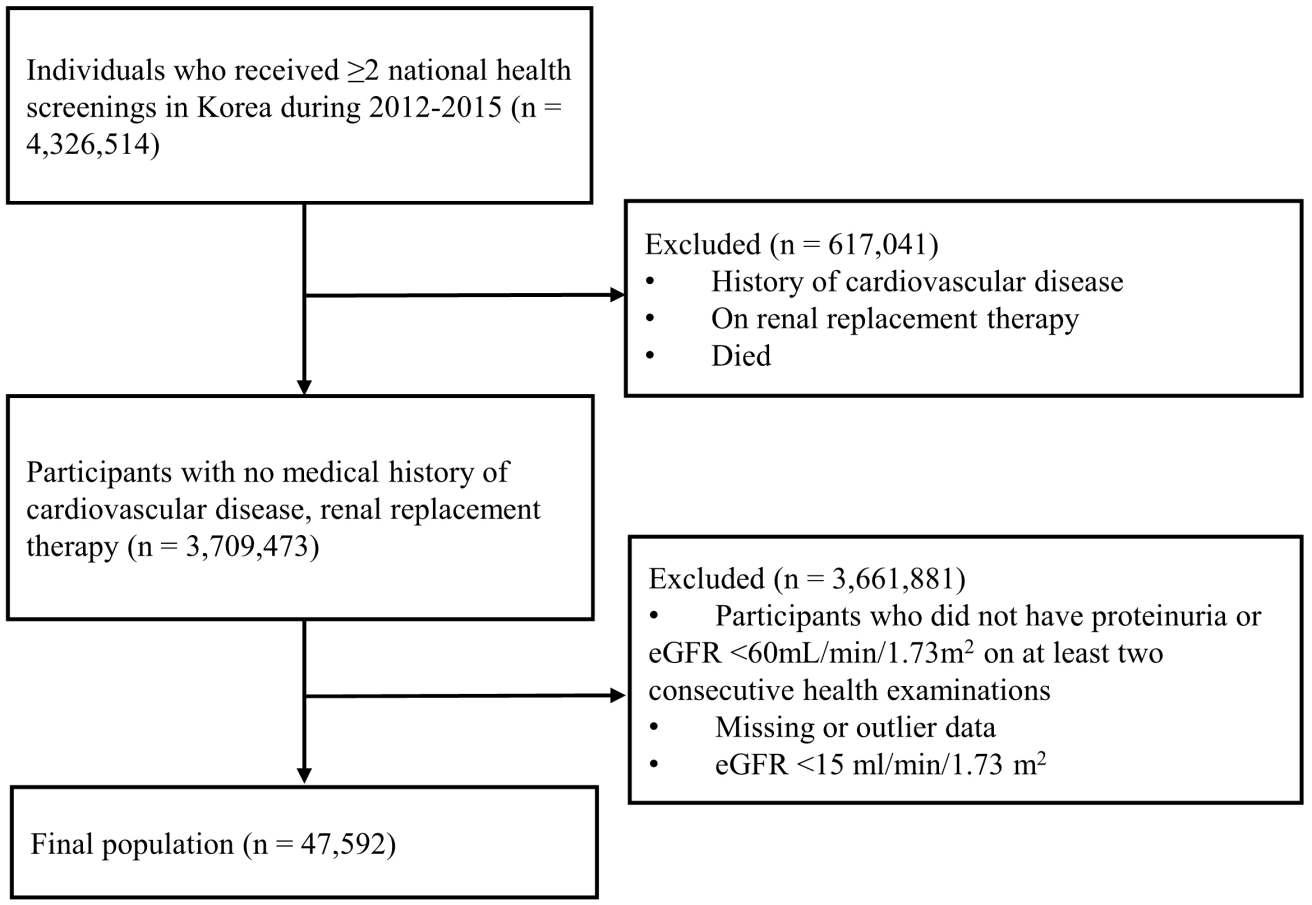
A flowchart of the study participant selection. eGFR, estimated glomerular filtration rate; BMI, body max index; CKD, chronic kidney disease.

Patients with CKD who participated in the National Health Screening between 2012 and 2015 were screened for inclusion. As the screening program is performed annually or biennially for all adults in South Korea, CKD was defined when either presence of proteinuria more than 1+ or eGFR less than 60mL/min/1.73m^2^ was consistently observed across at least two consecutive examinations. eGFR was calculated using the 2009 Chronic Kidney Disease Epidemiology Collaboration equation (CKD-EPI) (14). Exclusion criteria included individuals younger than 20 years, those with a history of ischemic heart disease (I20–25 in ICD-10 codes) or stroke (I60–69 in ICD-10 codes) at enrollment, those with eGFR less than 15mL/min/1.73m^2^, those receiving hemodialysis or peritoneal dialysis, and those with missing medical information. A total of 47,592 participants were included in the final analysis (**Figure 1**).

### 2.3 Data collection

Baseline demographic and clinical variables collected included age, sex, body mass index (BMI), height, weight, waist circumference, Charlson comorbidity index (CCI), CKD-EPI, systolic and diastolic blood pressure, fasting blood glucose, hemoglobin, total cholesterol, triglyceride, high density lipoprotein (HDL) and low density lipoprotein (LDL) cholesterol levels. The CCI was determined by calculating the score allocated to each comorbid condition using the ICD-10 diagnoses. Metabolic syndrome was defined as the presence of three or more of the following (13, 15): elevated blood pressure (≥130 mmHg systolic and/or ≥85 mmHg diastolic and/or use of antihypertensive drugs), hypertriglyceridemia (≥150 mg/dL or treatment for elevated triglycerides and/or use of lipid-lowering drugs), low high-density lipoprotein cholesterol (<40 mg/dL in men,<50 mg/dL in women), elevated fasting glucose (≥110 mg/dL and/or use of antidiabetic agents) or increased waist circumference (>90 cm for men, >80 cm in women). Self-reported lifestyle variables included smoking, alcohol consumption, and physical activity. Smoking status was classified as non-smoker, former smoker, or current smoker, and alcohol consumption was classified as 0 or 1 drinking per week, or drinking ≥ 2 times per week. Moderate physical activity was defined as exercise ≥ 3 times per week. All baseline data were obtained at the time when participants first fulfilled the CKD criteria and follow-up began thereafter.

### 2.4 TyG index

Participants were categorized into four groups based on their TyG index levels. The TyG index was calculated using the following formula (16);


$$\text{TyG index}=\text{ ln}\frac{\left[\text{triglyceride}\left(mg/dL\right)\right]\times \left[\text{fasting blood glucose}\left(mg/dL\right)\right]}{2}$$

The four quartiles based on TyG index levels were classified as follows; quartile 1,< 8.46; quartile 2, ≥ 8.46 and< 8.80; quartile 3, ≥ 8.80 and< 9.18; and quartile 4, ≥ 9.18.

### 2.5 Outcomes measures

The primary end points of this study were the occurrence of CV events, progression to ESKD, and all-cause mortality. CV events included myocardial infarction and ischemic stroke. Myocardial infarction and ischemic stroke were identified using ICD codes I20–25 and I60-69, respectively, which were newly prescribed during hospitalization or on at least two outpatient visits during the follow-up period. Progression to ESKD was defined as sustained requirement for hemodialysis, peritoneal dialysis, or renal transplantation. Participants who progressed to ESKD were identified by (i) specific insurance codes for renal replacement therapy to receive additional insurance coverage, (ii) procedure codes for renal replacement therapy prescribed for more than 3 months, and/or (iii) procedure or diagnostic codes for kidney transplantation. Subgroup analyses were performed based on age, sex, BMI, presence of metabolic syndrome, urine protein, and diabetes to account for diverse patterns of the TyG index associated with older age, obesity, kidney injury, glucose dysregulation, and metabolic dysfunction, all of which may affect clinical outcomes. Patients were followed up from January 1, 2016, until the date of death, last recorded checkup, or December 31, 2020, whichever occurred first.

### 2.6 Statistical analysis

Quantitative data were expressed as mean ± standard deviation, and categorical data were presented as numbers and percentages. Baseline characteristics and differences between groups were evaluated using chi-square tests and analysis of variance, as appropriate. Incident rates were calculated as the number of events per 1,000 person-years. The association between the TyG index and CV events, progression to ESKD, and all-cause mortality was analyzed using univariable and multivariable Cox regression models. Covariates in the multivariate Cox regression model included age, sex, BMI, smoking, drinking, physical activity, CCI, systolic blood pressure, eGFR, hemoglobin, LDL-cholesterol. Kaplan-Meier plots were generated to illustrate cumulative event rates for each clinical outcome. In the analysis treating exposures as a continuous variable, the multivariable-adjusted restricted cubic spline curves were constructed with three knots. In addition to the main effects in the fully adjusted models, interactions between the TyG index and subgroup characteristics or eGFR were evaluated. Statistical significance was defined as *P* value< 0.05. All statistical analyses were performed using SAS (version 9.4; SAS Institute, Cary, NC) and R (version 3.4.1; The R Foundation for Statistical Computing, Vienna, Austria; http://www.R-project.org).

## 3 Results

### 3.1 Baseline characteristics

Baseline demographics and clinical parameters across to TyG index quartiles are presented in **Table 1**. The mean TyG index was 8.10 ± 0.27 in quartile 1 (n = 11,900), 8.64 ± 0.12 in quartile 2 (n = 11,890), 9.04 ± 0.13 in quartile 3 (n = 11,904), and 9.75 ± 0.45 in quartile 4 (n = 11,898), with an overall mean of 8.88 ± 0.67 for the study population. Participants in quartile 4 were younger and included a higher proportion of males compared with those in the other quartiles. Higher TyG index quartiles were associated with increased BMI, greater waist circumference and greater prevalence of unfavorable lifestyle factors, including current smoking, frequent alcohol consumption, and physical inactivity. Blood pressure, fasting plasma glucose, and triglyceride level increased progressively with the rise of TyG index level.

**Table 1:** Baseline characteristics of the study population by the TyG index.

Characteristic	Triglyceride glucose index				*P*
	Quartile 1 (n = 11,900)	Quartile 2 (n = 11,890)	Quartile 3 (n = 11,904)	Quartile 4 (n = 11,898)	
Age (years)	62.7 ± 15.0	65.6 ± 13.4	64.8 ± 13.0	60.7 ± 13.3	<0.001
Male (%)	5,884 (49.5)	6,505 (54.7)	7,205 (60.5)	8,265 (69.5)	<0.001
BMI (kg/m^2^)	23.4 ± 3.2	24.6 ± 3.3	25.3 ± 3.3	26.1 ± 3.6	<0.001
Waist circumference (cm)	80.1 ± 9.2	84.1 ± 14.9	86.1 ± 8.6	88.5 ± 8.9	<0.001
Smoking					
Non-smoker (%)	8,354 (70.2)	7,776 (65.4)	6,999 (58.8)	5,798 (48.7)	<0.001
Ex-smoker (%)	2,238 (18.8)	2,497 (21.0)	2,810 (23.6)	2,999 (25.2)	
Current smoker (%)	1,308 (11.0)	1,617 (13.6)	2,095 (17.6)	3,101 (26.1)	
Alcohol consumption					
0, 1 days/week (%)	10,023 (84.2)	9,805 (82.5)	9,371 (78.7)	8,392 (70.5)	<0.001
≥2 days/week (%)	1,877 (15.8)	2,085 (17.5)	2,533 (21.3)	3,506 (29.5)	
Moderate physical activity (%)	7,342 (61.7)	7,090 (59.6)	6,763 (56.8)	6,438 (54.1)	<0.001
CCI	3.2 ± 2.5	3.4 ± 2.5	3.6 ± 2.5	3.8 ± 2.6	<0.001
eGFR (mL/min/1.73m^2^)	60.4 ± 21.9	57.1 ± 19.0	58.5 ± 20.2	64.0 ± 24.0	<0.001
SBP (mmHg)	125.1 ± 15.4	128.2 ± 15.3	130.0 ± 15.5	131.9 ± 15.7	<0.001
DBP (mmHg)	76.2 ± 10.2	77.6 ± 10.1	78.9 ± 10.2	80.5 ± 10.9	<0.001
Fasting glucose (mg/dL)	93.7 ± 14.1	101.3 ± 17.5	110.2 ± 25.5	142.5 ± 56.0	<0.001
Hemoglobin (g/dL)	13.2 ± 1.7	13.5 ± 1.8	13.8 ± 1.8	14.2 ± 1.9	<0.001
Total cholesterol	183.7 ± 36.4	191.6 ± 39.2	197.3 ± 41.2	207.4 ± 49.0	<0.001
Triglyceride (mg/dL)	73.3 ± 18.3	114.8 ± 20.8	160.5 ± 33.7	282.6 ± 158.0	<0.001
LDL-cholesterol (mg/dL)	110.7 ± 33.2	116.3 ± 35.9	116.6 ± 38.2	109.8 ± 43.4	<0.001

BMI, body mass index; CCI, Charlson comorbidity index; eGFR, estimated glomerular filtration rate; SBP, systolic blood pressure; DBP, diastolic blood pressure; LDL, low-density lipoprotein.

### 3.2 TyG index and risk of CV events, progression to ESKD, and all-cause mortality

**Figure 2:**
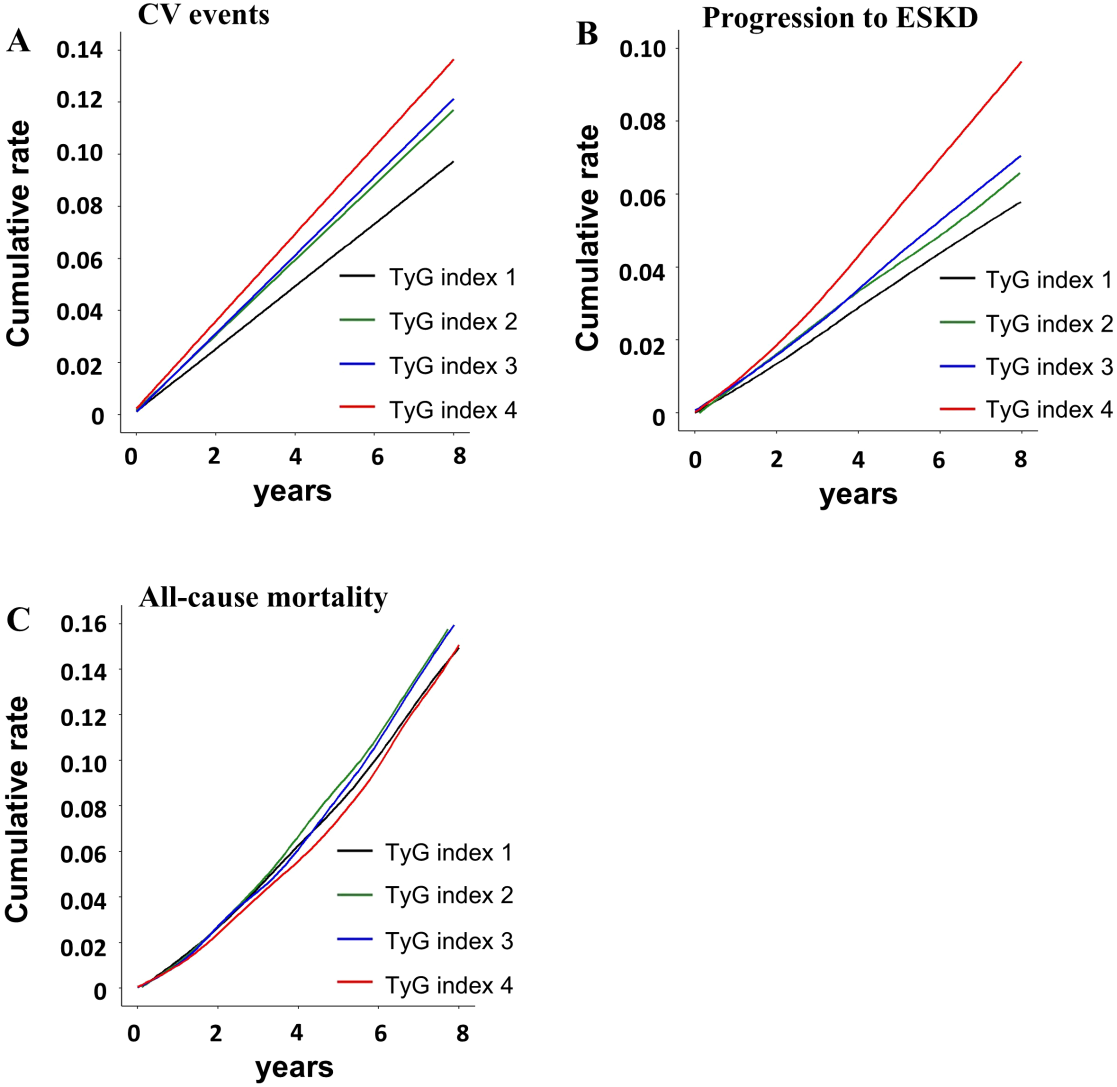
Cumulative event rates for adverse clinical outcomes according to TyG index: **(A)** cardiovascular (CV) events, **(B)** progression to end-stage kidney disease (ESKD), and **(C)** all-cause mortality. CV, cardiovascular; ESKD, end-stage kidney disease.

During a median follow-up of 90.8 ± 18.5 months, CV events and death were observed in 5,514 patients (11.6%), progression to ESKD in 3,475 patients (7.3%), and all-cause mortality in 7,502 patients (15.8%). The cumulative event rate of CV events varied significantly across TyG index quartiles, with the highest rate observed in quartile 4 (**Figure 2**). The cumulative event rate of progression to ESKD increased with higher TyG index quartiles.

**Table 2** shows the number of events, incidence rate in person-years, and hazard ratios (HRs) for all-cause mortality, CV events, and progression to ESKD. In univariate analyses, individuals in higher TyG index quartiles exhibited significantly greater risk of CV events and progression to ESKD. The association between the TyG index and risk of CV events remained significant after adjusting for multiple factors. Compared to TyG index quartile 1, the adjusted HRs (95% CI) for CV events were 1.08 (1.00 - 1.17), 1.13 (1.04 - 1.22), and 1.39 (1.28 - 1.51) for quartiles 2, 3, and 4, respectively. In a similar manner, a significant association was observed between the TyG index and the risk of progression to ESKD across all TyG index quartiles, that the risk increasing in higher quartiles as follows: adjusted HRs with 95% CI as 1.22 (1.08 - 1.38) in quartile 2, 1.35 (1.18 - 1.54) in quartile 3, 1.86 (1.65 - 2.10) in quartile 4. The risk of all-cause mortality was also significantly elevated in TyG index quartile 3 (adjusted HR 1.08, 95% CI 1.02 - 1.16) and 4 (adjusted HR 1.31, 95% CI 1.23 - 1.41). Additional adjustment models are provided in the **Supplementary Table 1**.

**Table 2:** Hazard ratios of all-cause mortality, composite of cardiovascular events, and progression to ESKD.

TyG index	Events, n	Incidence rate	Univariable analysis		Multivariable analysis					
					Model 1		Model 2		Model 3	
			HR (95% CI)	*P*	HR (95% CI)	*P*	HR (95% CI)	*P*	HR (95% CI)	*P*
CV events										
Quartile 1	1143	13.4	Reference	–	Reference	–	Reference	–	Reference	–
Quartile 2	1377	16.3	1.22 (1.13 – 1.32)	<0.001	1.12 (1.03 – 1.21)	0.005	1.10 (1.02 – 1.19)	0.020	1.08 (1.00 – 1.17)	0.491
Quartile 3	1435	17.0	1.27 (1.18 – 1.37)	<0.001	1.20 (1.11 – 1.29)	<0.001	1.15 (1.06 – 1.24)	<0.001	1.13 (1.04 – 1.22)	0.032
Quartile 4	1559	18.5	1.39 (1.29 – 1.50)	<0.001	1.53 (1.42 – 1.65)	<0.001	1.41 (1.30 – 1.53)	<0.001	1.39 (1.28 – 1.51)	<0.001
Progression to ESKD										
Quartile 1	680	7.8	Reference	–	Reference	–	Reference	–	Reference	–
Quartile 2	795	9.3	1.18 (1.06 – 1.30)	0.002	1.22 (1.10 – 1.35)	<0.001	1.30 (1.17 – 1.44)	<0.001	1.22 (1.08 – 1.38)	0.001
Quartile 3	854	9.9	1.26 (1.14 – 1.40)	<0.001	1.27 (1.15 – 1.41)	<0.001	1.37 (1.24 – 1.53)	<0.001	1.35 (1.18 – 1.54)	<0.001
Quartile 4	1146	13.4	1.72 (1.56 – 1.89)	<0.001	1.57 (1.42 – 1.73)	<0.001	1.63 (1.47 – 1.81)	<0.001	1.86 (1.65 – 2.10)	<0.001
All-cause mortality										
Quartile 1	1786	20.1	Reference	–	Reference	–	Reference	–	Reference	–
Quartile 2	1960	22.2	1.11 (1.04 – 1.18)	0.002	0.96 (0.90 – 1.02)	0.171	0.98 (0.92 – 1.05)	0.596	1.00 (0.93 – 1.06)	0.920
Quartile 3	1953	22.0	1.10 (1.03 – 1.17)	0.005	1.03 (0.96 – 1.09)	0.451	1.05 (0.99 – 1.12)	0.127	1.08 (1.02 – 1.16)	0.017
Quartile 4	1803	20.2	1.01 (0.94 – 1.07)	0.870	1.29 (1.20 – 1.37)	<0.001	1.26 (1.17 – 1.34)	<0.001	1.31 (1.23 – 1.41)	<0.001

**Figure 3:**
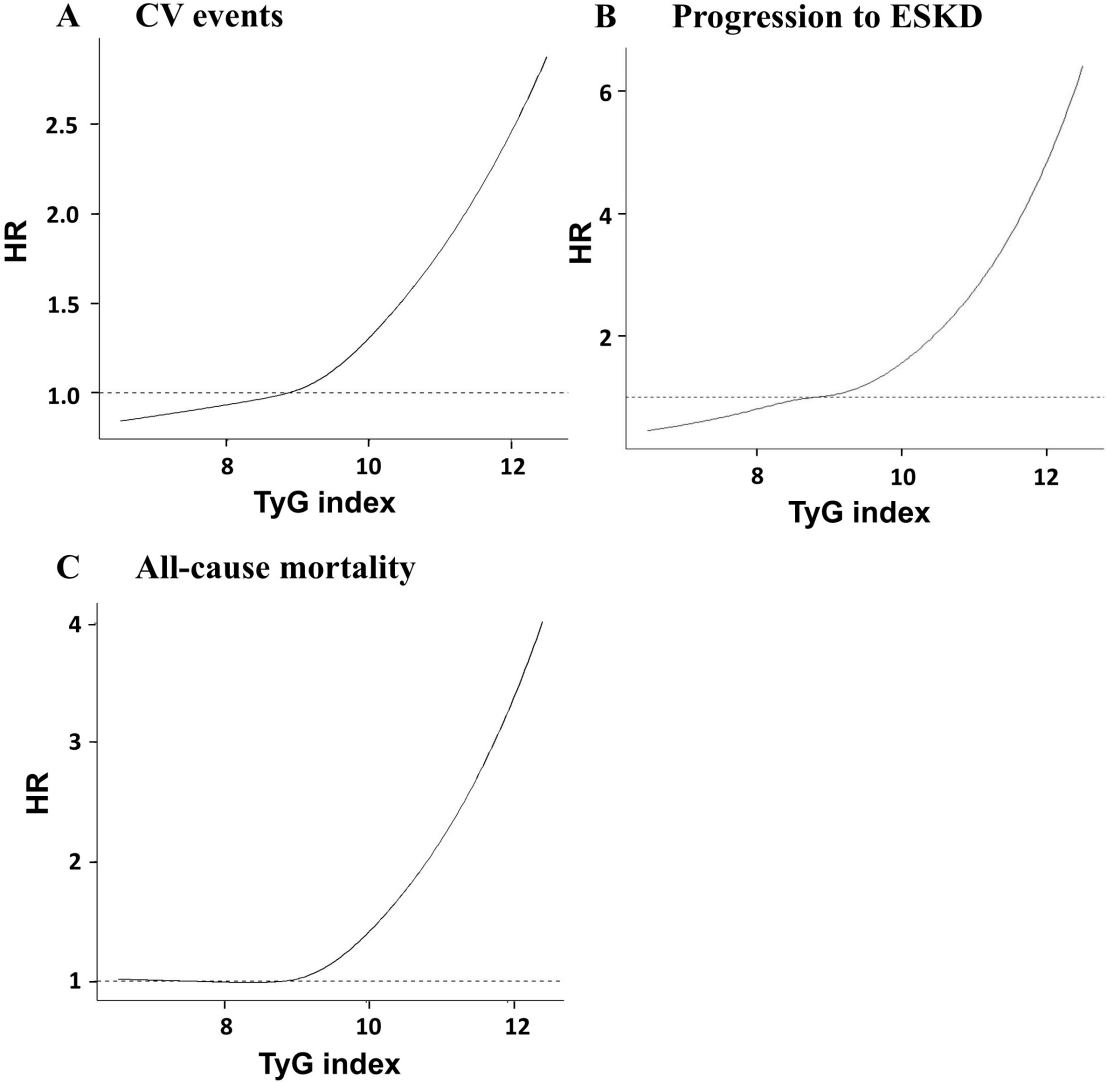
Association between the TyG index and the risk of adverse clinical outcomes: (A) cardiovascular (CV) events, (B) progression to end-stage kidney disease (ESKD), and (C) all-cause mortality. CV, cardiovascular; ESKD, end-stage kidney disease; HR, hazard ratio.

Restricted cubic spline curves indicated a continuous association between the TyG index and HRs for CV events, progression to ESKD, and all-cause mortality (**Figure 3**). The HRs for CV events and progression to ESKD increased with higher TyG index values, with risk accelerating at elevated levels. Meanwhile, the risk of all-cause mortality increased markedly only at higher TyG index values, with no significant changes observed at lower values.

### 3.3 Subgroup analysis

Pre-specified subgroup analysis based on age, sex, BMI, urine protein, diabetes, and metabolic syndrome are presented in **Table 3**. Risks of CV events, progression to ESKD, and all-cause mortality generally increased with each TyG index quartile increment across all subgroups. A more pronounced increase in HRs for CV events was observed in patients aged< 65 years (P for interaction<0.001), those with proteinuria (P for interaction<0.001), and those with diabetes (P for interaction = 0.003) compared to patients aged ≥ 65 years, those without proteinuria and those without diabetes. The risk of progression to ESKD was greater in participants with proteinuria than in those without proteinuria (P for interaction = 0.001). Similarly, the increase in all-cause mortality risk was more prominent in participants aged< 65 years and those with diabetes or metabolic syndrome. Significant interactions were observed between the TyG index and predefined subgroup characteristics.

**Table 3:** Pre-specified subgroup analysis on the risk of adverse clinical outcomes based on TyG index quartiles.

Characteristic	CV events		Progression to ESKD		All-cause mortality	
	Adjusted HR (95% CI)	*P* for interaction	Adjusted HR (95% CI)	*P* for interaction	Adjusted HR (95% CI)	*P* for interaction
Age<65 years	1.38 (1.28 – 1.48)	<0.001	1.51 (1.41 – 1.62)	0.374	1.21 (1.11 – 1.32)	<0.001
Age ≥65 years	1.19 (1.12 – 1.26)		1.33 (1.20 – 1.46)		1.13 (1.08 – 1.18)	
Male	1.32 (1.24 – 1.39)	0.246	1.49 (1.39 – 1.60)	0.804	1.19 (1.13 - 1.25)	0.552
Female	1.19 (1.10 – 1.29)		1.46 (1.32 – 1.62)		1.20 (1.12 – 1.29)	
BMI<25 kg/m^2^	1.29 (1.22 – 1.38)	0.258	1.45 (1.34 – 1.56)	0.800	1.16 (1.11 – 1.22)	0.073
BMI ≥ 25 kg/m^2^	1.26 (1.17 – 1.35)		1.49 (1.37 – 1.63)		1.23 (1.15 – 1.31)	
Urine protein ≤ trace	1.19 (1.12 – 1.26)	<0.001	1.20 (1.09 – 1.33)	0.001	1.14 (1.08 – 1.19)	0.002
Urine protein ≥ 1+	1.41 (1.31 – 1.51)		1.42 (1.32 – 1.52)		1.25 (1.17 – 1.34)	
No diabetes	1.11 (1.03 – 1.20)	0.003	1.39 (1.24 – 1.56)	0.152	1.07 (1.00 – 1.15)	<0.001
Diabetes	1.29 (1.21 – 1.36)		1.44 (1.35 – 1.54)		1.25 (1.19 – 1.32)	
No metabolic syndrome	1.21 (1.10 – 1.32)	0.880	1.53 (1.34 – 1.75)	0.187	1.08 (1.01 – 1.17)	<0.001
Metabolic syndrome	1.23 (1.15 – 1.30)		1.29 (1.20 – 1.38)		1.27 (1.20 – 1.34)	

### 3.4 Association between TyG index, kidney function, and adverse clinical outcomes

**Figure 4:**
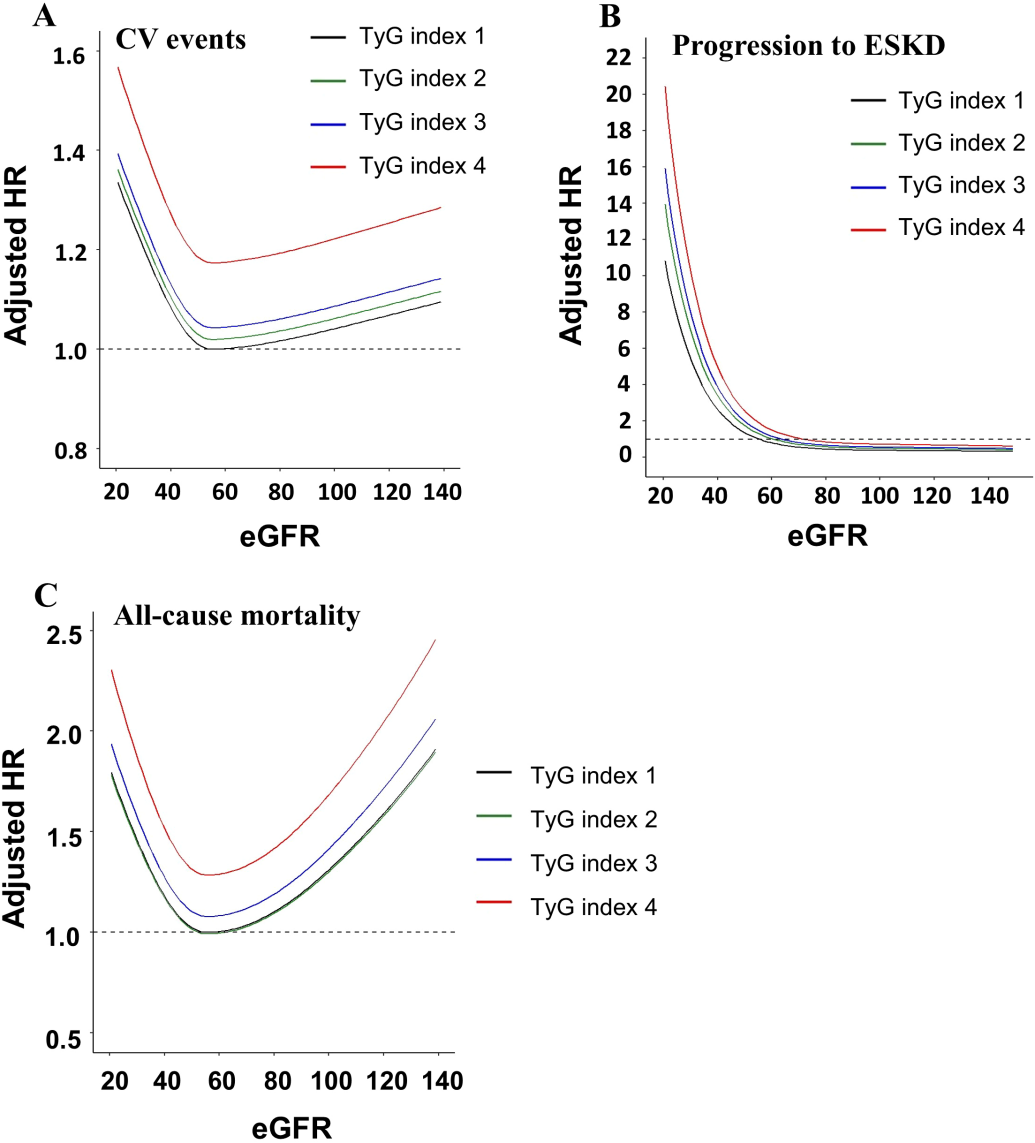
Association between estimated glomerular filtration rate (eGFR) and adverse clinical outcomes according to TyG index quartiles: **(A)** cardiovascular (CV) events, **(B)** progression to end-stage kidney disease (ESKD), and **(C)** all-cause mortality. eGFR, estimated glomerular filtration rate; CV, cardiovascular; ESKD, end-stage kidney disease; HR, hazard ratio.

To evaluate whether the relationship between kidney function and adverse clinical outcomes differed by TyG index, the risks of CV events, progression to ESKD, and all-cause mortality were evaluated across eGFR levels stratified by TyG index quartiles using spline curves (**Figure 4**). A J-shaped relationship was observed between eGFR and CV event risk, while progression to ESKD followed an L-shaped pattern and all-cause mortality exhibited a U-shaped association. The shape of the curve for CV events remained consistent across TyG index quartiles, suggesting a relatively uniform association (P for interaction = 0.616). However, the increase in ESKD risk with declining eGFR was more pronounced in CKD patients with higher TyG index quartiles (P for interaction< 0.001). Similarly, the interaction between TyG index and eGFR in relation to all-cause mortality was statistically significant, indicating a stronger association in higher TyG index quartiles (P for interaction< 0.001).

## 4 Discussion

This nationwide observational study evaluated the clinical implications of the TyG index in patients with CKD. The highest TyG index quartile was associated with the greatest risk of CV events, progression to ESKD, and all-cause mortality, after adjusting for multiple covariates. The risk of these adverse outcomes increased progressively with higher TyG index values, with a more pronounced acceleration at elevated levels. Furthermore, the association between a higher TyG index and the risk of adverse clinical outcomes was more pronounced under different risk profiles, and a synergistic interaction between the TyG index and eGFR was observed in relation to the risk of ESKD progression and mortality. These findings indicate a strong association between the TyG index and major adverse clinical outcomes in patients with CKD, supporting its utility as a reliable marker for identifying high-risk groups.

The elevated risk of CV events in patients with CKD is attributed to a combination of traditional and CKD-specific risk factors (17). CKD-specific factors, including mineral bone disorders, uremic toxins, and low-grade inflammation, are recognized as significant contributors to CV outcomes (18). Consequently, traditional risk factors for CV events may be considered less influential in patients with CKD compared with the general population (19). Some studies have even suggested that dyslipidemia or hyperglycemia may play a relatively minor role in CV risk in this population (20, 21). Nevertheless, our study clearly demonstrated a significant association between the TyG index and CV risk in patients with CKD. These findings suggest that the TyG index serves as a valuable marker for assessing CV risk in patients with CKD, highlighting the relevance of traditional risk factors even in the context of CKD.

Previous studies have reported that hyperinsulinemia triggers glomerular hyperfiltration, endothelial dysfunction, and increased vascular permeability, all of which adversely affect renal hemodynamics (22). In addition, insulin resistance and elevated triglyceride levels contribute to the progression of proteinuria through disruptions in insulin signaling and lipid metabolism at the molecular level, ultimately leading to kidney failure (23, 24). Although these biological mechanisms are established, clinical evidence supporting their role in ESKD is limited. To address this knowledge gap, we investigated the association between the TyG index and the risk of ESKD. Our study demonstrated that higher TyG index quartiles were significantly associated with an increased risk of ESKD in patients with CKD, with the risk rising sharply at higher TyG index levels. These findings highlight the pathophysiological significance of insulin resistance in CKD progression and provide clinical validation of its biological link to renal deterioration.

Previous studies have reported an association between a higher TyG index and increased all-cause mortality, with varying strengths of association (10, 25). Consistent with these reports, our study observed a significantly elevated risk of all-cause mortality in individuals in the highest TyG index quartile after adjusting for multiple covariates, whereas the association was not significant in the univariate analysis. Sensitivity analyses using different adjustment sets suggested that this discrepancy was largely attributable to adjustment for BMI. In CKD populations, higher BMI has sometimes been associated with lower mortality risk; accounting for BMI may attenuate this protective effect (13, 26), making the adverse association between a higher TyG index and all-cause mortality more apparent. In addition, we also observed a differential pattern in the association between continuous TyG index levels and adverse event risks. While the risk of CV events and progression to ESKD increased consistently across all TyG index levels, the risk of all-cause mortality remained relatively stable at lower TyG index levels and increased only at higher values. These findings suggest that lower TyG index levels may have limited prognostic value for all-cause mortality, while remaining clinically meaningful for CV events and kidney failure. Therefore, our results highlight that the prognostic utility of the TyG index in patients with CKD may be outcome-specific, emphasizing the need for outcome-centered interpretation in clinical practice.

Subgroup analysis revealed specific conditions associated with an increased risk of adverse clinical outcomes. Patients aged< 65 years or those with proteinuria or diabetes exhibited a higher risk of CV events and all-cause mortality compared to their counterparts. Additionally, the presence of metabolic syndrome was significantly associated with an increased risk of all-cause mortality. These findings suggest that the clinical significance of the TyG index is greater in patients with multiple CV risk factors, highlighting the need for risk profile–based assessment. Furthermore, patients with proteinuria had a greater risk of ESKD progression than those without proteinuria. This suggests that insulin resistance may exert a synergistic effect in accelerating renal deterioration when underlying kidney damage is already present (27).

The modifying effect of the TyG index on the association between eGFR and the risk of adverse clinical outcomes was also evaluated. We found that the association between a higher TyG index and the risk of ESKD progression was more pronounced in CKD patients with lower baseline eGFR. These findings suggest that insulin resistance may contribute to faster renal deterioration, predominantly in patients with reduced baseline kidney function. Notably, although the TyG index did not modify the association between eGFR and CV events, it enhanced the link between eGFR and all-cause mortality. These findings suggest that the modifying role of the TyG index in the association between eGFR and adverse clinical outcomes may differ based on the type of outcome. This further implies a potential involvement of insulin resistance in the increased risk of non-cardiovascular mortality.

There were several limitations to this study. First, as this was an observational study based on the NHIS database, not all confounding variables were fully accounted for, potentially introducing bias. Second, selection bias may be present, as the study population may not fully include the entire Korean population. Individuals in better health are more likely to receive health screening examinations, whereas those with severe comorbidities may be underrepresented. Finally, because this study was conducted in a Korean population, the results may not be fully generalizable to other populations. The distribution and clinical significance of the TyG index can be different based on the degree and pattern of obesity, which are known to differ across ethnicities and regions. Therefore, caution is needed when extending these findings to populations with different genetic backgrounds or lifestyle characteristics.

## 5 Conclusions

In conclusion, this study demonstrated that a higher TyG index is significantly associated with an increased risk of CV events, progression to ESKD, and all-cause mortality in patients with CKD, and it significantly interacted with eGFR in determining the risks of ESKD and mortality. These findings suggest that the TyG index is a valuable marker for assessing the risk of major adverse outcomes in patients with CKD. Incorporating the TyG index into clinical practice may improve risk stratification and facilitate the development of targeted management strategies to reduce the burden of CKD-related complications.

## Data Availability

The data analyzed in this study is subject to the following licenses/restrictions: The raw data were generated at the Health Insurance Review and Assessment Service. The authors cannot distribute the data without permission from this organization. To access the database, a study proposal outlining the purpose, design, and duration of the analysis must be submitted to the Health Insurance Review and Assessment Service through their website: https://www.hira.or.kr. Requests to access these datasets should be directed to https://www.hira.or.kr.

## Glossary

TyG index: Triglyceride glucose index
CV: Cardiovascular
CKD: Chronic kidney disease
ESKD: End-stage kidney disease
eGFR: Estimated glomerular filtration rate
HOMA-IR: Homeostatic model assessment for insulin resistance
NHID: National Health Information Database
NHIS: National Health Insurance Service
ICD: International Classification of Diseases
CKD-EPI: Chronic Kidney Disease Epidemiology Collaboration equation
BMI: Body mass index
CCI: Charlson comorbidity index
HDL: High density lipoprotein
LDL: Low density lipoprotein
