# Supplementary material for: Predictive value of the triglyceride-glucose index for adverse clinical outcomes in chronic kidney disease
Source: Front Endocrinol (Lausanne). 2026 Jan 21;16:1656800. doi: 10.3389/fendo.2025.1656800 (PMC12867898; doi:10.3389/fendo.2025.1656800)
Supplement: Supplementary file 1 [file Table1.docx]

**Supplementary table 1.** Additional multivariable models evaluating the hazard ratios of all-cause mortality, composite of cardiovascular events, and progression to ESKD

|  |  |  | Univariable analysis | | Multivariable analysis | | | | | | | | | |
| --- | --- | --- | --- | --- | --- | --- | --- | --- | --- | --- | --- | --- | --- | --- |
|  |  |  |  |  | Model 1 | | Supplementary model S1 | | Supplementary model S2 | | Model 2 | | Model 3 | |
|  | Events, n | Incidence rate | HR (95% CI) | *P* | HR (95% CI) | *P* | HR (95% CI) | *P* | HR (95% CI) | *P* | HR (95% CI) | *P* | HR (95% CI) | *P* |
| CV events | | |  |  |  |  |  |  |  |  |  |  |  |  |
| Quartile 1 | 1143 | 13.4 | Reference | - | Reference | - | Reference | - | Reference | - | Reference | - | Reference | - |
| Quartile 2 | 1377 | 16.3 | 1.22  (1.13 – 1.32) | <0.001 | 1.12  (1.03 – 1.21) | 0.005 | 1.12  (1.03 – 1.21) | 0.007 | 1.10  (1.02 – 1.20) | 0.015 | 1.10  (1.02 – 1.19) | 0.020 | 1.08  (1.00 – 1.17) | 0.491 |
| Quartile 3 | 1435 | 17.0 | 1.27  (1.18 – 1.37) | <0.001 | 1.20  (1.11 – 1.29) | <0.001 | 1.19  (1.10 – 1.29) | <0.001 | 1.17  (1.08 – 1.26) | <0.001 | 1.15  (1.06 – 1.24) | <0.001 | 1.13  (1.04 – 1.22) | 0.032 |
| Quartile 4 | 1559 | 18.5 | 1.39  (1.29 – 1.50) | <0.001 | 1.53  (1.42 – 1.65) | <0.001 | 1.53  (1.41 – 1.65) | <0.001 | 1.47  (1.35 – 1.59) | <0.001 | 1.41  (1.30 – 1.53) | <0.001 | 1.39  (1.28 – 1.51) | <0.001 |
| Progression to ESKD | | |  |  |  |  |  |  |  |  |  |  |  |  |
| Quartile 1 | 680 | 7.8 | Reference | - | Reference | - | Reference | - | Reference | - | Reference | - | Reference | - |
| Quartile 2 | 795 | 9.3 | 1.18  (1.06 – 1.30) | 0.002 | 1.22  (1.10 – 1.35) | <0.001 | 1.33  (1.19 – 1.47) | <0.001 | 1.33  (1.20 – 1.48) | <0.001 | 1.30  (1.17 – 1.44) | <0.001 | 1.22  (1.08 – 1.38) | 0.001 |
| Quartile 3 | 854 | 9.9 | 1.26  (1.14 – 1.40) | <0.001 | 1.27  (1.15 – 1.41) | <0.001 | 1.45  (1.30 – 1.61) | <0.001 | 1.47  (1.32 – 1.63) | <0.001 | 1.37  (1.24 – 1.53) | <0.001 | 1.35  (1.18 – 1.54) | <0.001 |
| Quartile 4 | 1146 | 13.4 | 1.72  (1.56 – 1.89) | <0.001 | 1.57  (1.42 – 1.73) | <0.001 | 1.85  (1.67 – 2.06) | <0.001 | 1.91  (1.72 – 2.12) | <0.001 | 1.63  (1.47 – 1.81) | <0.001 | 1.86  (1.65 – 2.10) | <0.001 |
| All-cause mortality | |  |  |  |  |  |  |  |  |  |  |  |  |  |
| Quartile 1 | 1786 | 20.1 | Reference | - | Reference | - | Reference | - | Reference | - | Reference | - | Reference | - |
| Quartile 2 | 1960 | 22.2 | 1.11  (1.04 – 1.18) | 0.002 | 0.96  (0.90 – 1.02) | 0.171 | 1.00  (0.94 – 1.07) | 0.966 | 0.99  (0.93 – 1.05) | 0.699 | 0.98  (0.92 – 1.05) | 0.596 | 1.00  (0.93 – 1.06) | 0.920 |
| Quartile 3 | 1953 | 22.0 | 1.10  (1.03 – 1.17) | 0.005 | 1.03  (0.96 – 1.09) | 0.451 | 1.11  (1.04 – 1.18) | 0.002 | 1.07  (1.01 – 1.15) | 0.033 | 1.05  (0.99 – 1.12) | 0.127 | 1.08  (1.02 – 1.16) | 0.017 |
| Quartile 4 | 1803 | 20.2 | 1.01  (0.94 – 1.07) | 0.870 | 1.29  (1.20 – 1.37) | <0.001 | 1.43  (1.34 – 1.53) | <0.001 | 1.35  (1.27 – 1.45) | <0.001 | 1.26  (1.17 – 1.34) | <0.001 | 1.31  (1.23 – 1.41) | <0.001 |

Abbreviations: HR, hazard ratio; CI, confidence interval; CV, cardiovascular; ESKD, end-stage kidney disease; eGFR, estimated glomerular filtration rate

Incidence rate was represented as 1,000 person-years.

Multivariable analyses were adjusted for the following covariates:

Model 1 : age, sex

Supplementary model S1 : Model 1 plus BMI

Supplementary model S2 : Supplementary model S1 plus smoking, drinking, physical activity

Model 2 : Model 1 plus BMI, smoking, drinking, physical activity, Charlson comorbidity index

Model 3 : Model 2 plus systolic blood pressure, eGFR, hemoglobin, LDL-cholesterol
